# Overall survival following stereotactic radiosurgery for ten or more brain metastases: a systematic review and meta-analysis

**DOI:** 10.1186/s12885-023-11452-7

**Published:** 2023-10-19

**Authors:** Hamoun Rozati, Jiarong Chen, Matt Williams

**Affiliations:** 1 London Gamma Knife Centre, Platinum Medical Centre, Wellington Hospital, Lodge Road, London, UK; 2 Computational Oncology Group, Department of Surgery and Cancer, Imperial College London, London, UK; 3 Clinical Experimental Center, Jiangmen Key Laboratory of Clinical Biobanks and Translational Research, Jiangmen Central Hospital, Jiangmen, 529030 China; 4 Department of Radiotherapy, Charing Cross Hospital, Imperial College Healthcare NHS Trust, London, UK

**Keywords:** Stereotactic radiosurgery, Radiotherapy, Multiple metastases

## Abstract

**Background:**

Brain metastases are the most common intracranial tumours. Variation exists in the use of stereotactic radiosurgery for patients with 10 or more brain metastases. Concerns include an increasing number of brain metastases being associated with poor survival, the lack of prospective, randomised data and an increased risk of toxicity.

**Methods:**

We performed a systematic review and meta-analysis to assess overall survival of patients with ten or more brain metastases treated with stereotactic radiosurgery as primary therapy. The search strings were applied to MEDLINE, Embase and the Cochrane Central Register of Controlled Trials (CENTRAL). Log hazard ratios and standard errors were estimated from each included study. A random-effects meta-analysis using the DerSimonian and Laird method was applied using the derived log hazard ratios and standard errors on studies which included a control group.

**Results:**

15 studies were included for systematic review. 12 studies were used for pooled analysis for overall survival at set time points, with a predicted 12 month survival of 20–40%. The random-effects meta-analysis in five studies of overall survival comparing ten or greater metastases against control showed statistically worse overall survival in the 10 + metastases group (1.10, 95% confidence interval 1.03–1.18, p-value = < 0.01, *I*^*2*^ = 6%). A funnel plot showed no evidence of bias. There was insufficient information for a meta-analysis of toxicity.

**Discussion:**

Overall survival outcomes of patients with ten or more brain metastases treated with SRS is acceptable and should not be a deterrent for its use. There is a lack of prospective data and insufficient real-world data to draw conclusions on toxicity.

**PROSPERO ID:**

CRD42021246115

**Supplementary Information:**

The online version contains supplementary material available at 10.1186/s12885-023-11452-7.

## Key points

Patients with 10 or more brain metastases treated with stereotactic radiosurgery can have prolonged survival outcomes.

There is a lack of prospective trials to inform on treatment decisions.

**Supplementary Information:**

The online version contains supplementary material available at 10.1186/s12885-023-11452-7.

## Importance of the study

This systematic review and meta-analysis is the first of its kind in the literature and provides information on overall survival outcomes and toxicities encountered in patients with ten or more brain metastases treated with stereotactic radiosurgery. Centres treating patients with ten or more brain metastases are doing so based only on retrospective real-world data analyses, the vast majority of which are from single centres and single radiotherapy platforms. This review provides an additional evidence resource for practitioners of stereotactic radiosurgery to aide in the management of this difficult patient group. The methods used to predict survival outcomes through the calculation of log hazard ratios and standard errors allowed analysis of small, retrospective case series. To our knowledge, this is the first meta-analysis of this patient group gives evidence for acceptable overall survival outcomes post-treatment, and provides further evidence for the use of stereotactic radiosurgery for these patients.

Overall survival following stereotactic radiosurgery for ten or more brain metastases: a systematic review and meta-analysis.

**Supplementary Information:**

The online version contains supplementary material available at 10.1186/s12885-023-11452-7.

## Introduction

Brain metastases (BMs) are the most common intracranial tumours in adults. One population-based study over 28 years found primary malignant sites with the highest incidence proportions of BMs were lung (19.9%), melanoma (6.9%), renal (6.5%) and breast cancers (5.1%) [1]. However, BMs may arise from any malignancy and although their exact incidence is not known, they are thought to account for more than half of all intracranial tumours, with an incidence up to 40,000 per annum in the United States [2]. Management of brain metastases is dependent on a multitude of factors. Surgery is considered most appropriate for patients with severe mass effect or impending herniation and is also considered in patients with oligometastatic disease as a potentially curative approach. Radiotherapy is considered a standard approach for patients without evidence of severe mass effect or where multiple metastases are found. While historically whole brain radiotherapy (WBRT) was the preferred radiation method of choice, it is being supplanted by stereotactic radiosurgery (SRS) for many cases where there are a limited number of brain metastases. SRS is the delivery of a set number of doses of radiation delivered to a small target. This is achieved using many, non-coplanar radiation beans which converge to a single point. Healthy tissue is protected from the high doses of radiation by the steep drop-off in dose which occurs away from the intended target volume. While novel systemic anti-cancer agents with good brain penetrance may change this treatment algorithm, further studies are required to determine if they can replace local therapies for the management of BMs. SRS is now considered a standard option without adjuvant WBRT for “limited” metastases, although the definition of “limited” varies across the literature.

Prospective, randomised data has provided evidence for the use of SRS alone for cases of 1–4 BMs [3–5]. Based on data such as the prospective, observational JLGK0901 study [6] which demonstrated non-inferiority in treating 5–10 BMs as compared to 1–4 BMs, a number of guidelines have eliminated the set BM number criterion for determining eligible patients for SRS [7, 8]. However, significant discrepancies exist in the use of SRS in patients with 10 or greater metastases. While the Congress of Neurological Surgeons guidelines recommend more than 4 metastases can be treated with SRS up to a volume of 7 cc, this is only given a level 3 recommendation [9]. The American Radium Society’s guidelines in the management of 11–20 BMs resulted in no recommendation that SRS could be used alone [10], and the latest American Society for Radiation Oncology (ASTRO) guidelines also does not recommend SRS for more than 10 BMs [11]. Concerns may be due to an increasing number of BMs being associated with poor survival [12], the lack of prospective, randomised data and the increased risk of toxicities such as radiation necrosis when treating multiple targets with SRS. The aim of this systematic review and meta-analysis was to collate the available literature in the use of SRS as primary therapy in patients with 10 or more BMs and evaluate resulting overall survival. We also aimed to collect information on rates of radiation-induced complications, in particular rates of radiation necrosis.

## Methods

### Search strategies and selection criteria

We performed a systematic review and meta-analysis to assess overall survival of patients with ten or more brain metastases treated with SRS as primary therapy. The study was prospectively registered with PROSPERO (CRD42021246115) and performed in accordance to the Preferred Reporting Items for Systematic Reviews and Meta-Analysis (PRISMA) checklist [13]. Search terms were devised by HR in conjunction with a medical librarian from Imperial College London. Patients with ten or more brain metastases treated with stereotactic radiotherapy alone were included. The full inclusion and exclusion criteria, and the search strings used, are available in the appendix in Sect. 1.1 and 1.2 respectively.

The search strings were applied to MEDLINE, Embase and the Cochrane Central Register of Controlled Trials (CENTRAL). Searches were restricted to human studies available in English, and relevant grey literature. Trial registries were also searched. Studies published from 1st January 1960 to 1st April 2021 were included and transferred to Covidence (www.covidence.org) for analysis. Abstract screening and full-text eligibility were performed independently by HR and JC. Disputes were resolved by the senior author (MW).

### Data analysis

Data extraction was performed by HR. If different studies involving the same patient cohort was found, meta-analysis was performed only with the study with the largest cohort to reduce bias. All studies were included for narrative synthesis.

Total number of patients in the study, the number of patients with 10 or greater metastases, the proportion of primary malignancies, the median number of metastases in the multi-metastases group, the median survival, the median follow up time, the median cumulative volume of metastases treated and the number of patients alive at follow up were collated. The main summary measure was proportion of patients alive at set time points, with a primary outcome of overall survival. Survival outcomes were extracted where documented in the study manuscript or were otherwise derived from survival curves. Information on rates of radiation necrosis were also collected where available.

Log hazard ratios and standard errors were estimated from each included study using the methods and spreadsheet as developed by Tierney, et al. [14]. All studies with sufficient information available underwent a pooled proportion analysis via a generalised linear mixed-effects model to create an estimated pooled survival curve of all included studies at set time points (3, 6, 9, 12, 15, 18 and 24 months). Heterogenicity was assessed using the *I*^*2*^ test. P values < 0.05 were considered statistically significant. As significant levels of heterogeneity were anticipated between the studies analysed, a random-effects meta-analysis using the DerSimonian and Laird method was applied using the derived log hazard ratios and standard errors on studies which included a control group. A funnel plot was generated as a measure of reporting bias.

Statistical analysis was performed using R version 4.1.0, and packages tidyverse, meta and dmetar were utilised. A bias analysis was conducted using the Risk Of Bias In Non-randomised Studies - of Interventions (ROBINS-I) tool [15]. A summary of the evidence was rated using the GRADE system [16]. The certainty of evidence for overall survival was assessed by considering the risk of bias in the studies, inconsistency, imprecision, indirectness, and the possibility for publication bias.

## Results

**Fig. 1:**
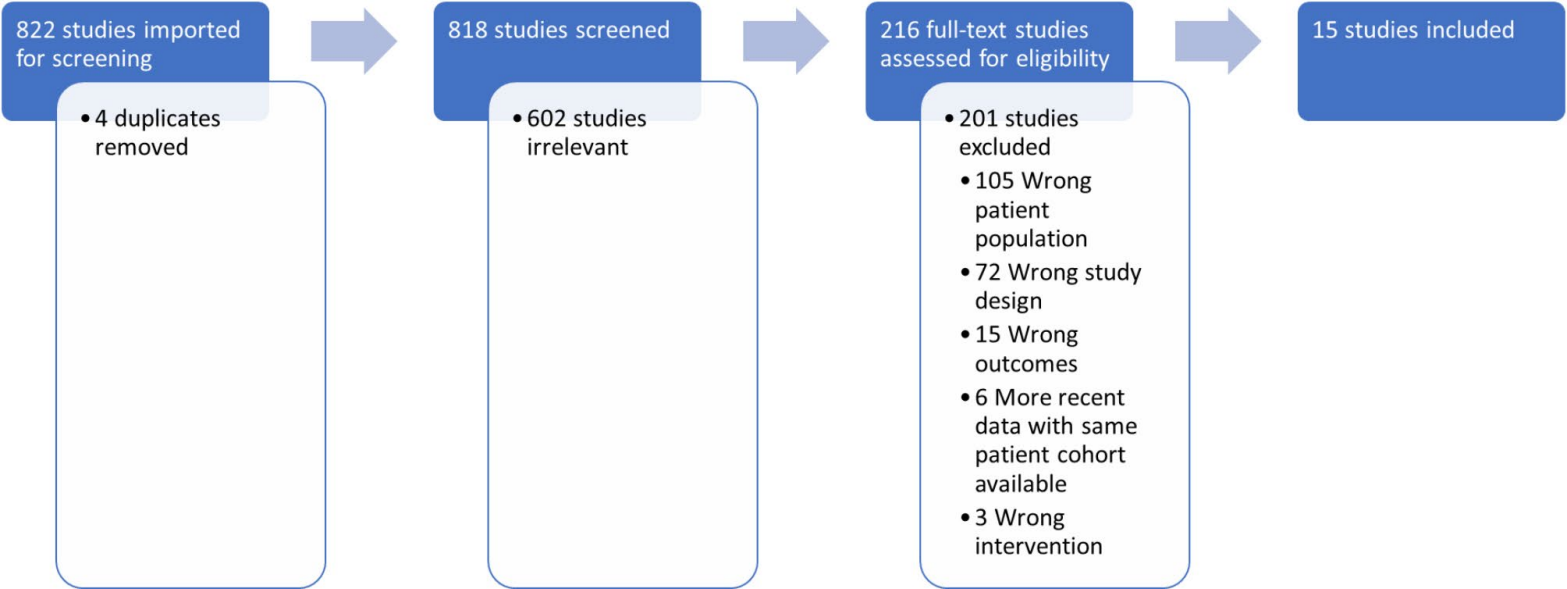
Study selection performed on the Covidence online platform as per the PRISMA guidelines. Studies were searched for in Embase, MEDLINE and CENTRAL databases. Screening performed by two authors (HR and JC) with disputes resolved by senior author (MW)

Our database search yielded 822 studies, from which 4 duplicate studies were removed. Abstract and full text screening was performed as shown in Fig. 1, culminating in 15 studies which were included in the systematic review.

A summary of the included studies for narrative synthesis is shown in Table 1. The total number of treated patients with ten or more BMs was 2360, with one of the included studies not documenting the number of 10 + metastases patients. Six studies included centres based in the United States, six in Japan, two in the Republic of Korea, one in Italy and one in Australia. Most included patients had a primary malignancy of lung cancer (1424), breast cancer (343), and melanoma (133). Median overall survival from all studies was 8.15 months (interquartile range 2.5-9.875). Studies included patients treated from 1994 to 2018. All except one study was conducted on the Gamma Knife, with one study (Minniti, et al. 2020) using linear accelerator technology. Median cumulative tumour volume treated was not reported in every study but of those presented ranged from 0.38 to 9.98 cc.

All included studies were retrospective case series. Six of the fifteen studies compared patients with ten or more metastases against a control group; one control group was WBRT in ten or more metastases (Mizuno 2019) and so was not included in the meta-analysis, the remaining five studies were patients with fewer than ten metastases and so were included. 12 of 15 studies were included in the pooled analysis of survival outcomes. Of the three excluded studies, Susko, et al. 2020 and Yamamoto, et al. 2021 had overlapping data with Ali et al., 2017, and Serizawa, et al. 2006 did not specify the number of patients with more than ten metastases.

**Table 1:** Summary of studies included in systematic review following screening process

	Centre, country	Follow up (months)	No. of patients included	Period patients undergoing treatment	Primary cancer sites include (%)	No. of patients metastases in 10 + group	Median number of mets in multi-mets group	Control group included?	Median survival in multi-metastases group (months)	Median survival in limited metastases group (months)	Cumulative volume (cm^3^)	SRS platform used	No. of patients alive at the end of follow up	Rates of AREs documented?	Included in meta-analysis?
Ali 2017 [17]	University of California, USA Gamma Knife House, Japan Tokyo Gamma Knife Centre, Japan	6.4 (median)	5750	1994–2014	Breast (12.3) GI (12.0) Lung (65.0) Melanom (4.9) RCC (5.5)	981	Not specified	Yes (2–9 metastases)	5.5	6.4	Breast 6.90 GI 9.17 Lung 3.75 Melanoma 1.99 RCC 5.35	Gamma Knife	None	Not specified	Yes
Bowden 2019 [18]	Pittsburgh, USA	Not specified	93	Dec 2008 – Dec 2017	Breast (34.8) Lung (42.4) Melanoma (22.8)	93 (15 + mets)	Breast: 23 Lung: 21 Melanoma: 21	No	Breast 18.0 Lung 9.4 Melanoma 6.3	n/a	Breast 8.75 Lung 6.89 Melanoma 9.98	Gamma Knife	Not specified	Not specified	No – no control group
Chang 2010 [19]	Yonsei University, Republic of Korea	6 (mean) 0–44 (range)	323	Oct 2005 – Oct 2008	NSCLC (39) Breast (12.4) RCC (8.7) CRC (8.4) SCLC (4.6) Gynae (3.4) HCC (2.5) Thyroid (1.5) H&N (1.5) Prostate (1.2)	50 total (17 (11–15 mets group) 33 (> 15 mets group))	Not specified	Yes (1–5 metastases)	13.0 (11–15 mets group) 8.0 33 (> 15 mets group)	10.0 (1–5 metastases group)	Not specified	Gamma Knife	Not specified	ARE in 23 pts (7.12%). Only 1 pts (3.0%) in > 15 mets group	Yes
Ehrlich 2019 [20]	Zucker School of Medicine at Hofstra/Northwel, USA	Not specified	55	Mar 2014 – Apr 2018	NSCLC (47%) Breast (22%) Melanoma (16%) Other (15%)	40 (synchronously treated)	10–15 mets: 32 pts 16–20 mets: 9 pts > 20 mets: 14 pts	No	9.5	n/a	0.3167–54.86	Gamma Knife	Not specified	Not specified	No – no contorl group
Izard 2019 [21]	Macquarie University Hospital, Australia	11 (0–62.4 months)	180	Aug 2010 – Jul 2017	NSCLC (27%) Breast (27) Melanoma (24) Other (22)	47	11–20 mets: 30 pts > 20 mets: 17 patients	Yes (4–9 mets)	8.5 months	6 months	Median: 0.57 (range < 0.005–5.44)	Gamma Knife	39	Long-term radionecrosis in 22 pts (12.2%)	Yes
Kim 2008 [22]	Sungkyunkwan University School of Medicine, Seoul, Korea	Not specified	26	Aug 2002 – Dec 2007	NSCLC (80) Breast (12) Unknown (8)	26	16.6	No	7.82	n/a	10.9 cc (1.0-42.2)	Gamma Knife	8	Not specified	No – no control group
Minniti 2020 [23]	University of Siena, Italy	10.8 (median)	40	Jan 2017 – Dec 2018	Lung (42.5%) Melanoma (25%) Breast (17.5%) Kidney (15)	40	13	No	14.1	n/a	0.38 Range 0.17–6.8	LINAC	21	Imaging suggestive of necrosis in 14 pts (35%). Grade 2 or 3 in 7 pts (14.5%)	No – no control group
Mizuno 2019 [24]	Komaki City Hospital, Japan	6.7 (SRS group) 6.4 (WBRT group)	44	Jan 2009 – Dec 2016	NSCLC (100%)	24	20	Yes	7.3	7.2	Not specified	Gamma Knife	Not specified	Not specified	Yes
Nakazaki 2013 [25]	Ota Memorial Hospital; Chiba University; Tsukiji Neurologic Clinic, Japan	4.0 (median) 0.5–21.2 (range)	47	Jan 1999 – Mar 2011	SCLC (100%)	17	30	Yes (1–10 mets)	5	9	Not specified	Gamma Knife	5	No AREs reported in any pts included in study	Yes
Raldow 2013 [26]	Yale School of Medicine, USA	Not specified	103	2000–2010	Melanoma (33%) NSCLC (32%) Breast (17) RCC (6) SCLC (5) Others (7)	18	84	Yes (5–10 mets)	8.3	7.6	0.33–34.6	Gamma Knife	Not specified	Not specified	Yes
Rava 2013 [27]	Tufts Medical Center Boston, USA	7.5 (median)	53	2004–2010	NSCLC (38) Breast (34) Others (28)	53	10.9	No	6.5	n/a	Not specified	Gamma Knife	Not specified	Not specified	No – no control group
Serizawa 2006 [28]	Chiba University, Japan	Not specified	1127	Not specified	Lung (69.8%) GI (12.3) Breast (6.6) Urinary (5) Others (6.5)	Not specified	Not specified	Yes (1–4 metastases)	10	15	0.8 range 0.1–24	Gamma Knife	Not specified	AREs not noted in any pts	No – missing no of 10 + mets patients. Data overlaps with Nakazawa 2013
Susko 2020 [29]	University of California, USA	7.4 (2.7–15.9)	143	Mar 1999 – Dec 2016	Breast (36.4) NSCLC (34.3) Melanoma (21.0) Other (8.3)	143	13	No	11.7	n/a	4.1 (IQR, 2.0-9.9)	Gamma Knife	6	16 pts (11%) with ARE on imaging. 3 pts (2%) symptomatic	No – no control group, overlap with Ali 2017
Suzuki 2000 [30]	Shin-Koga Hospital Gamma Knife Cente, Japan	3.0 (0 -8.7)	24	Jul 1998 – Jan 2000	Lung (83) Breast (12.5) CRC (4.5)	24	20	No	2.5	n/a	0.66 (range 0.003–18.4 )	Gamma Knife	12	Not specified	No – no control group
Yamamoto 2021 [31]	Katsuta Hospital Mito Gamma House; Ibaraki Tokyo Gamma Unit Center, Japan	15.5 (IQR 1.3–22.8)	1515	1998–2018	NSCLC (59) Breast (13.1) SCLC (11.2) GI (7.9) Kidney (2.2) Other (6.6)	804	14 (12–17)	Yes (5–10 mets)	6.5	7.7	54% < 7 46% ≥ 7	Gamma Knife	174	Not specified	No -overlap with Ali 2017

**Fig. 2:**
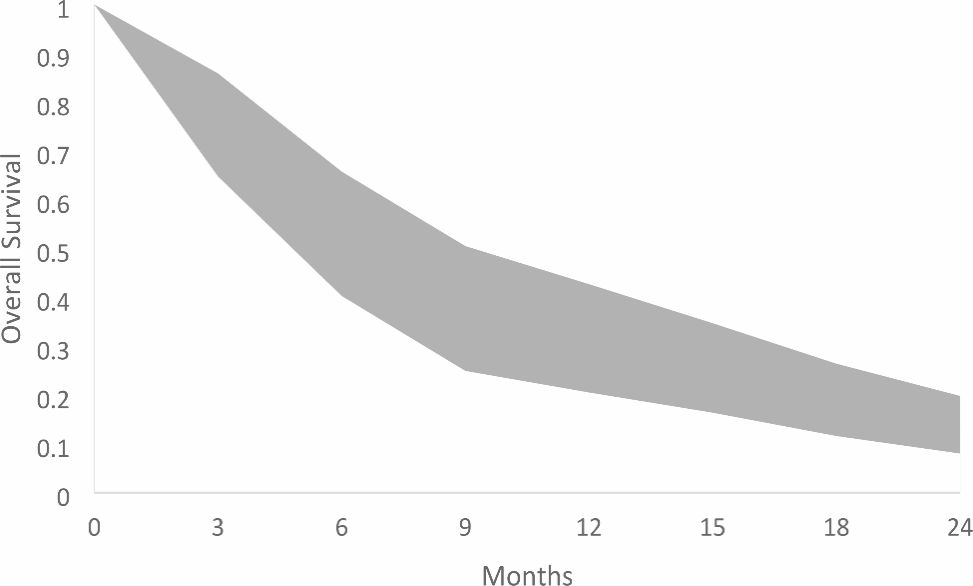
Confidence band chart of pooled overall survival outcomes created through the lower and upper confidence intervals presented in Table 2

ARE: adverse radiation effect; IQR: interquartile range; LINAC: linear accelerator; mets: metastases; NSCLC: non-small cell lung cancer; pts: patients; RCC: renal cell carcinoma. Twelve studies (80% of all studies) were used for pooled analysis for overall survival at set time points. The summary table and resulting confidence band chart is shown in Table 2; Fig. 2 respectively. There was significant study heterogenicity at each of the calculated time points (*I*^*2*^ range 73.9–84.4%) which is why a confidence band chart is used to visual the data, highlighting the uncertainty in the results. Raw data for each time point is available in the appendix (Sect. 1.3)

**Table 2:** Summary of pooled survival outcomes at set time points formed by a linear mixed-effects model

Month	Survival	L-CI	U-CI	*I* ^*2*^
3	0.7699	0.6478	0.8589	76.0
6	0.5326	0.4031	0.6579	73.9
9	0.3682	0.2496	0.5053	81.1
12	0.3051	0.2054	0.4273	83.9
15	0.2442	0.1640	0.3473	84.4
18	0.1786	0.1162	0.2644	79.3
24	0.1283	0.0804	0.1984	81.2

L-CI: lower bound of 95% confidence interval; U-CI: upper bound of 95% confidence interval

**Fig. 3:**
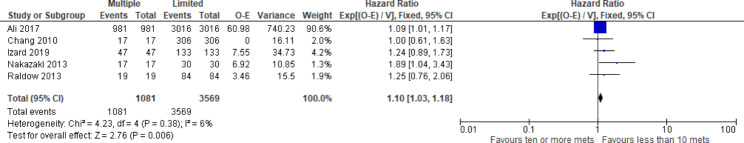
Random-effects meta-analysis of ten or greater metastases against control, showing a statistically significant trend for improved overall survival outcomes in the fewer than 10 brain metastases group

The random-effects meta-analysis of ten or greater metastases against control groups is shown in Fig. 3. Five studies including a control group were used. The pooled hazard ratio of survival favoured the control group, and reached statistical significance with high levels of homogeneity between studies (1.10, 95% confidence interval 1.03–1.18, p-value = < 0.01, *I*^*2*^ = 6%). The study by Ali, et al. contributed a significant weight to the meta-analysis, although their analysis found no statistically significant difference in survival between the 2–9 and 10 + BM groups [17]. The corresponding funnel plot did not demonstrate evidence of bias and is shown in the appendix (Sect. 1.4).

There was only limited data available on rates of radiation necrosis and so was deemed unsuitable for meta-analysis. Rates of radiation necrosis found on imaging in patients treated with Gamma Knife ranged from 0 to 12.2%. The study by Chang, et al. 2010 specified necrosis in 3% of treated patients with 15 or greater metastases, and no treated patients with 11–14 metastases. The remaining studies did not distinguish rates of necrosis between those patients with 10 or greater metastases and those with fewer. No studies used histological determination for confirmation of radiation necrosis, and instead relied on radiologically-suspicious features. The single linear accelerator-based study (Minniti, et al. 2020) reported necrosis in 35% of patients treated, but only half of these were symptomatic and required treatment (57% required medical therapy only, 43% required surgery).

All studies were assessed for bias, and a summary is presented in Table 3. Three of the included studies demonstrated serious risks of bias as per the ROBINS-I criteria.

**Table 3:** ROBINS-I analysis to assess the risk of bias in each included study

	Bias due to confounding	Bias in selection of participants into the study	Bias in classification of intervention	Bias due to deviations from intended interventions	Bias due to missing data	Bias in measurement of outcomes	Bias in selection of the reported result	Overall risk of bias
Ali 2017	2	1	1	1	1	1	2	Moderate
Bowden 2019	2	1	1	1	1	1	2	Moderate
Chang 2010	2	1	1	1	1	1	2	Moderate
Erlich 2019	2	1	1	1	1	1	1	Moderate
Izard 2019	2	1	1	1	1	1	1	Moderate
Kim 2008	2	1	1	1	1	1	1	Moderate
Minniti 2020	2	2	1	1	1	1	1	Moderate
Mizuno 2019	3	1	1	1	1	1	1	Serious
Nakazaki 2013	3	2	1	1	1	1	1	Serious
Raldow 2013	2	1	1	1	1	1	1	Moderate
Rava 2013	2	1	1	1	1	1	1	Moderate
Serizawa 2006	2	1	1	1	1	1	1	Moderate
Susko 2020	2	1	1	1	1	1	1	Moderate
Suzuki 2000	2	1	1	1	1	1	1	Serious
Yamamoto 2021	1	1	1	1	1	1	1	Moderate

0 = no information/unclear, 1=“Low risk”, 2=“Moderate risk”, 3=“Serious risk”, 4=“Critical risk” of bias. The quality of evidence for assessing overall survival in patients with 10 or more BMs is shown in Table 4. The quality of evidence was downgraded to very low as per the GRADE criteria due to limitations in study design and the risk of bias, particularly in the largest studies included for analysis

**Table 4:** Summary of findings for overall survival outcomes

			Certainty of evidence (GRADE)					
Outcome	Study population and study design	Summary effect	Methodological limitations	Inconsistency	Indirectness	Imprecision	Publication bias	Overall quality
Overall survival	15 studies (2360 patients)	Overall survival of patients treated with SRT for 10 + BMs is comparable to patients with 1–9 BMs	Critical	Not serious	Not serious	Critical	Not serious	Very low ^a^

^a^downgraded due to the small, non-randomised retrospective nature of studies without incorporating confounding factors for survival. The relatively high confidence intervals within studies also strongly suggests imprecision

## Discussion

This systematic review and meta-analysis collated the available data on overall survival of patients with ten or more BMs treated with SRS. Our results demonstrate that overall survival in patients with 10 or more BMs treated with SRS is slightly worse than patients with 2–9 BMs [12]. However, a median survival of 8.15 months for patients with ten or more BMs is more impressive when considered that many of the patients were treated before 2010, and therefore before the widespread use of intracranially active systemic anti-cancer treatments. Some patients were treated as early as 1994, although unfortunately no breakdown of when individual patients were treated is available.

Our pooled survival analysis allowed the construction of a survival curve for the reader to gauge survival outcomes in patients with ten or more BMs. For example, the 24-month survival 95% confidence interval of 0.08–0.20 months is likely greater than most treating physicians may predict and may inform treatment decisions for this patient group. The visual representation of long-term survival in this patient cohort and the review dedicated to patients with ten or more BMs has not been previously published.

Our review highlights the lack of prospective, randomised data to inform treatment decisions for patients with ten or greater BMs. Our pooled random effects meta-analysis comparing ten or more BMs to a control group showed statistically significantly worse overall survival in the 10 + group compared to the < 10 BMs group, and the corresponding *I*^*2*^ test showed low heterogeneity between studies. The meta-analysis was dominated by the largest study by Ali, et al. [17] but whereas their study did not demonstrate a statistically significant difference in overall survival between high and low BM groups, the meta-analysis presented here does show a statistically significant difference. However we do not believe this should deter the use of SRS in patients with 10 or more BMs. The lower limit of the 95% confidence interval is close to unity (1.03), and confounding factors such as total volume of intracranial disease and primary malignancy have not been corrected for. The corresponding funnel plot demonstrates a low risk of publication bias. In light of the lack of prospective, randomised data, our review suggests non-inferiority for overall survival in patients with ten or more BMs.

A review of this nature will not provide a definitive answer to predict survival outcomes in this patient group. We believe the two most significant omissions in the current literature are firstly the lack of prospective, randomised data, and secondly the lack of detail in patient factors which impact on survival as well as SRS, including but not limited to: detailed cancer histology including mutation status; preceding, concurrent, and proceeding systemic anti-cancer therapy; and preceding and proceeding local intracranial therapy besides SRS, namely surgery and WBRT. This data is lacking in the studies included in this review.

Although RCTs would represent an ideal evidence base, and there are several randomised trials currently recruiting patients with multiple BMs and randomising between SRS and WBRT (NCT02953717, NCT03075072 and NCT03550391), there have been previous issues with trials of this nature. A Dutch randomised control trial of 4–10 BMs closed early due to a failure to accrue sufficient patients [32] and the North American Gamma Knife Consortium trial (NCT01731704) which had planned to randomise patients with 4–10 BMs treated with SRS against WBRT has also closed early. A further randomised controlled trial of patients with non-melanoma primaries and 4–15 BMs has been reported in abstract form, but has only 36 patients in each treatment arm, thus limiting interpretation of their results [33]. Other studies have reported outcomes from treating patients with multiple metastases, but in a staged form [34], and planning studies have confirmed that patients treated with SRS receive a low hippocampal radiotherapy dose [35].

A reason for the failure for randomised trials to accrue sufficient patients may be due to the lack of equipoise which exists in treating radiation oncologists and neurosurgeons. A patterns of care survey performed amongst the German Society for Radiation Oncology (DEGRO) found that WBRT is the preferred treatment modality in most multiple BMs cases over SRS. In an example case of a patient with NSCLC, 15 BMs and a prognosis over 6 months found that 89.1% of 165 respondents favoured WBRT over SRS [36]. Similarly, a patterns of care survey in the United States of 116 radiation oncologists found 82.5% of respondents report using WBRT for ten or more BMs over SRS [37]. A survey of practitioners allied to a radiosurgical society were however more likely to utilise SRS for patients with 7 or more metastases [38].This suggests that there may be a discrepancy between physicians with easy and regular access to dedicated SRS equipment. There may be an ingrained approach some physicians have towards treating patients with multiple BMs, and this may limit the numbers of patients referred for randomised trials.

The treatment paradigm for patients with multiple BMs is changing in light of the increasing number of novel systemic anti-cancer agents with good brain penetrance. Patients with oncogenic driver mutations such as epidermal growth factor receptor (EGFR) mutations or anaplastic lymphoma kinase (ALK) rearrangements may derive significant benefit in treating their intracranial disease from oral systemic therapy. This may avoid or delay the need for local intracranial treatment. There are a number of benefits in trying to avoid local therapies in this patient group. Intracranial tumours have shown significant response rates when targeted agents against these driver mutations are used. Patients with these driver mutation are living longer, and this increased survival will allow more time for side effects to develop from local therapies [39, 40]. Their use in lieu of local therapies, particularly in asymptomatic patients, is likely to increase. In the absence of prospective, randomised controlled data, studies need to include detailed information regarding the tumour mutation status and systemic anti-cancer therapy history of their patient cohort to adjust for their impact on overall survival.

We were unable to perform the planned meta-analysis on rates of radiation necrosis due to information on their incidence only being included in five studies. The disparate nature they were reported meant they were included for narrative synthesis only. Nonetheless, rates of necrosis detected on imaging were low, particularly in patients treated with Gamma Knife. Brain necrosis is also notorious difficult to diagnose and differentiate from disease progression using imaging alone. It is not specified whether the patients included in these studies would have undergone a biopsy to confirm necrosis, although this is not common practice in many centres. Rates of true necrosis may therefore not correlate well to those documented. The relatively low rates of necrosis across the studies presented provide assurance to treating physicians managing patients with ten or more metastases. Adverse radiation effects are difficult to accurately report in trials as they can occur more than ten years following radiation exposure [41]. This may be mitigated by studies demonstrating superior normal brain dosimetry for SRS over hippocampal-sparing WBRT in patients with 10 to 30 BMs [42]. Based on planning studies, SRS appears to deliver little hippocampal dose even in patients with multiple metastases [35]. Other studies have looked at multiple cumulative BMs which included toxicity data [34], but this may not correlate closely with single session SRS.

To reduce the impact of bias in this review we assessed each included study with design-specific criteria as set out by Viswanathan, et al. [43]. We also only included the largest cohort when data overlapped with more than one study. Nonetheless, the studies included are at significant risk of participation and attrition bias. The studies also showed high levels of heterogeneity in pooled survival analysis, likely due in part to the variations in primary malignancies, systemic therapies cancer genetics and comorbidities. Our GRADE assessment showed high levels of discrepancies in patient populations, size of effects and publication bias, and this is consistent with many systematic reviews of retrospective case series. Our statistical analysis is also limited by not having access to individual patient data, and instead basing analysis on aggregates derived from variable outcome measures reporting in each individual study. Interpretation of the results presented should be considered in this context. A further issue is the likelihood of overlap between patient cohorts from different studies. The cohorts as presented by Nakazaki [25] may have overlapping patients with the cohorts published by Serizawa and Yamamoto [28, 31]. However as only the Nakazaki cohort was included in the meta-analysis, and they only contributed 47 patients, we believe this is unlikely to significantly affect the results of our analysis.

We have not made any corrections for different treatment platforms, and have considered the most commonly used platforms of LINACs, Cyberknife and Gamma Knife as equivalent. While dosimetric data exists suggesting a difference in coverage and dose spill, no randomised prospective trials have shown a different in outcomes, whether tumour response or complication rates [44]. Advancements in the ability to deliver SRT with LINACs, coupled with their comparative availability, will likely increase its use for SRT in the future. Given much of the current data is based on the Gamma Knife platform, future studies will need to assess whether a change in platform leads to different outcomes in survival and tumour response.

In conclusion, this systematic review and meta-analysis provides insight into the survival outcomes of patients treated with SRS for ten or more BMs. Outcomes are similar to those published for unselected patients treated with SRS for more than one BM, downplaying the importance of number of BMs in patient survival. A meta-analysis of five studies demonstrated a significant survival difference in patients with ten or more BMs treated with SRS versus control, a finding not seen when reviewing the largest single analysis alone. However the lower limit of the 95% confidence interval was close to unity and data is likely to be effected by confounding factors for survival such as volume of disease. Our data suggests SRS is a suitable option for selected patients with ten or more BMs. Future work would ideally be in the form of a randomised, controlled clinical trial. Observational data requires more extensive detail on the primary malignancy, on additional intracranial and systemic treatments received, and on rates of radiation necrosis.

### Electronic supplementary material

Below is the link to the electronic supplementary material.

Supplementary Material 1

## Data Availability

All data generated or analysed during this study are included in this published article [and its supplementary information files].
