# Supplementary material for: Overall survival following stereotactic radiosurgery for ten or more brain metastases: a systematic review and meta-analysis
Source: BMC Cancer. 2023 Oct 19;23:1004. doi: 10.1186/s12885-023-11452-7 (PMC10585836; doi:10.1186/s12885-023-11452-7)

Appendix:

- 1. **Inclusion and exclusion criteria**

Inclusion criteria:

Patients with ten or more brain metastases treated with stereotactic radiotherapy alone.

Prior irradiation or surgery is allowed, as is fractionated and staged treatments.

Concurrent systemic therapy is also allowed.

Studies can use any stereotactic radiotherapy modality

Exclusion criteria:

Concurrent whole brain radiotherapy, leptomeningeal disease, metachronous treatment of ten or more metastases.

Dosimetric studies without clinical follow up.

- 1. **Search strings**

Ovid MEDLINE(R) ALL <1946 to April 23, 2021>

1 Radiosurgery.mp. [mp=title, abstract, original title, name of substance word, subject heading word, floating sub-heading word, keyword heading word, organism supplementary concept word, protocol supplementary concept word, rare disease supplementary concept word, unique identifier, synonyms]

2 (radiosurg* or stereotactic or linear accelerator or linac or cyberknife or gamma knife).mp. [mp=title, abstract, original title, name of substance word, subject heading word, floating sub-heading word, keyword heading word, organism supplementary concept word, protocol supplementary concept word, rare disease supplementary concept word, unique identifier, synonyms]

3 1 or 2

4 exp Brain Neoplasms/

5 ((brain or cerebral or cerebellum) adj5 (tumor* or tumour* or neoplas* or cancer* or carcinoma* or malignan* or metast*)).mp. [mp=title, abstract, original title, name of substance word, subject heading word, floating sub-heading word, keyword heading word, organism supplementary concept word, protocol supplementary concept word, rare disease supplementary concept word, unique identifier, synonyms]

6 4 or 5

7 randomized.ab.

8 controlled clinical trial.pt.

9 randomized controlled trial.pt.

10 clinical trials as topic.sh.

11 randomly.ab.

12 trial.ti.

13 case-control studies/ or cohort studies/ or cross-sectional studies/

14 7 or 8 or 9 or 10 or 11 or 12 or 13

15 3 and 6

16 14 and 15

**1.3 RAW DATA FOR POOLED SURVIVAL ANALYSIS**

Details on meta-analytical method:

- Random intercept logistic regression model

- Maximum-likelihood estimator for tau^2

- Hartung-Knapp adjustment for random effects model

- Logit transformation

- Normal approximation confidence interval for individual studies

**Review: 3 Month Survival**

proportion 95%-CI

Ali_2017 0.7000 [0.6706; 0.7279]

Bowden_2019_Breast 0.9500 [0.7445; 0.9920]

Bowden_2019_Lung 0.9000 [0.6839; 0.9740]

Bowden_2019_Melanoma 0.6500 [0.4310; 0.8199]

Chang_2010_11-15_mets 0.8000 [0.5493; 0.9292]

Chang_2010_15_plus_mets 0.9000 [0.7427; 0.9656]

Ehrlich_2019 0.8500 [0.7300; 0.9224]

Izard_2019 0.7000 [0.5556; 0.8132]

Mizuno_2019 0.8000 [0.5953; 0.9158]

Nakazaki_2013 0.5000 [0.2787; 0.7213]

Raldow_2013 0.8000 [0.5576; 0.9270]

Suzuki_2000 0.7000 [0.4936; 0.8482]

Rava_2013 0.9000 [0.7858; 0.9567]

Kim_2008 0.2000 [0.0873; 0.3952]

Number of studies combined: k = 14

proportion 95%-CI

Random effects model 0.7699 [0.6478; 0.8589]

Quantifying heterogeneity:

tau^2 = 0.8277; tau = 0.9098; I^2 = 76.0% [59.7%; 85.7%]; H = 2.04 [1.57; 2.64]

Test of heterogeneity:

Q d.f. p-value Test

54.06 13 < 0.0001 Wald-type

76.97 13 < 0.0001 Likelihood-Ratio

**Review: 6 Month Survival**

proportion 95%-CI

Ali_2017 0.5000 [0.4688; 0.5312]

Bowden_2019_Breast 0.8500 [0.6434; 0.9468]

Bowden_2019_Lung 0.3900 [0.2101; 0.6058]

Bowden_2019_Melanoma 0.3000 [0.1442; 0.5215]

Chang_2010_11-15_mets 0.7500 [0.5002; 0.8999]

Chang_2010_15_plus_mets 0.7500 [0.5771; 0.8684]

Ehrlich_2019 0.6500 [0.5162; 0.7637]

Izard_2019 0.6000 [0.4556; 0.7289]

Mizuno_2019 0.6000 [0.3986; 0.7724]

Nakazaki_2013 0.2000 [0.0708; 0.4507]

Raldow_2013 0.7000 [0.4599; 0.8648]

Suzuki_2000 0.4929 [0.3039; 0.6839]

Rava_2013 0.5100 [0.3779; 0.6407]

Kim_2008 0.1200 [0.0401; 0.3080]

Number of studies combined: k = 14

proportion 95%-CI

Random effects model 0.5326 [0.4031; 0.6579]

Quantifying heterogeneity:

tau^2 = 0.6506; tau = 0.8066; I^2 = 73.9% [55.6%; 84.6%]; H = 1.96 [1.50; 2.55]

Test of heterogeneity:

Q d.f. p-value Test

49.73 13 < 0.0001 Wald-type

68.85 13 < 0.0001 Likelihood-Ratio

**Review: 9 Month Survival**

proportion 95%-CI

Ali_2017 0.3000 [0.2721; 0.3294]

Bowden_2019_Breast 0.7500 [0.5386; 0.8852]

Bowden_2019_Lung 0.2500 [0.1104; 0.4723]

Bowden_2019_Melanoma 0.2500 [0.1104; 0.4723]

Chang_2010_11-15_mets 0.7500 [0.5002; 0.8999]

Chang_2010_15_plus_mets 0.5000 [0.3357; 0.6643]

Ehrlich_2019 0.6000 [0.4666; 0.7201]

Izard_2019 0.4500 [0.3153; 0.5924]

Mizuno_2019 0.5000 [0.3100; 0.6900]

Nakazaki_2013 0.1500 [0.0445; 0.4005]

Raldow_2013 0.4000 [0.2061; 0.6312]

Suzuki_2000 0.1230 [0.0398; 0.3216]

Rava_2013 0.3000 [0.1924; 0.4354]

Kim _2008 0.0800 [0.0206; 0.2640]

Number of studies combined: k = 14

proportion 95%-CI

Random effects model 0.3682 [0.2496; 0.5053]

Quantifying heterogeneity:

tau^2 = 0.7530; tau = 0.8678; I^2 = 81.1% [69.3%; 88.4%]; H = 2.30 [1.81; 2.93]

Test of heterogeneity:

Q d.f. p-value Test

68.88 13 < 0.0001 Wald-type

78.49 13 < 0.0001 Likelihood-Ratio

**Review: 12 Month Survival**

proportion 95%-CI

Ali_2017 0.2000 [0.1761; 0.2262]

Bowden_2019_Breast 0.6300 [0.4221; 0.7988]

Bowden_2019_Lung 0.1800 [0.0673; 0.4006]

Bowden_2019_Melanoma 0.1100 [0.0305; 0.3266]

Chang_2010_11-15_mets 0.5000 [0.2787; 0.7213]

Chang_2010_15_plus_mets 0.3000 [0.1691; 0.4743]

Ehrlich_2019 0.4000 [0.2799; 0.5334]

Izard_2019 0.3000 [0.1868; 0.4444]

Minniti 2020 0.6500 [0.4923; 0.7805]

Mizuno_2019 0.3000 [0.1518; 0.5064]

Nakazaki_2013 0.0200 [0.0007; 0.3784]

Raldow_2013 0.4000 [0.2061; 0.6312]

Rava_2013 0.2200 [0.1284; 0.3507]

Number of studies combined: k = 13

proportion 95%-CI

Random effects model 0.3051 [0.2054; 0.4273]

Quantifying heterogeneity:

tau^2 = 0.5809; tau = 0.7622; I^2 = 83.9% [73.8%; 90.0%]; H = 2.49 [1.96; 3.17]

Test of heterogeneity:

Q d.f. p-value Test

74.36 12 < 0.0001 Wald-type

79.93 12 < 0.0001 Likelihood-Ratio

**Review: 15 Month Survival**

proportion 95%-CI

Ali_2017 0.1500 [0.1290; 0.1737]

Bowden_2019_Breast 0.6000 [0.3944; 0.7755]

Bowden_2019_Lung 0.1000 [0.0260; 0.3161]

Bowden_2019_Melanoma 0.1100 [0.0305; 0.3266]

Chang_2010_11-15_mets 0.4500 [0.2394; 0.6802]

Chang_2010_15_plus_mets 0.2500 [0.1316; 0.4229]

Ehrlich_2019 0.4000 [0.2799; 0.5334]

Izard_2019 0.3000 [0.1868; 0.4444]

Mizuno_2019 0.2500 [0.1169; 0.4564]

Nakazaki_2013 0.0200 [0.0007; 0.3784]

Raldow_2013 0.2000 [0.0730; 0.4424]

Susko_2020 0.4000 [0.2958; 0.5141]

Rava_2013 0.1700 [0.0909; 0.2955]

Number of studies combined: k = 13

proportion 95%-CI

Random effects model 0.2442 [0.1640; 0.3473]

Quantifying heterogeneity:

tau^2 = 0.4767; tau = 0.6904; I^2 = 84.4% [74.9%; 90.4%]; H = 2.53 [2.00; 3.22]

Test of heterogeneity:

Q d.f. p-value Test

77.10 12 < 0.0001 Wald-type

81.53 12 < 0.0001 Likelihood-Ratio

**Review: 18 Month Survival**

proportion 95%-CI

Ali_2017 0.1000 [0.0827; 0.1204]

Bowden_2019_Breast 0.4500 [0.2646; 0.6504]

Bowden_2019_Lung 0.0600 [0.0104; 0.2788]

Bowden_2019_Melanoma 0.1100 [0.0305; 0.3266]

Chang_2010_11-15_mets 0.4000 [0.2017; 0.6376]

Chang_2010_15_plus_mets 0.2000 [0.0963; 0.3697]

Ehrlich_2019 0.2500 [0.1533; 0.3803]

Izard_2019 0.3000 [0.1868; 0.4444]

Mizuno_2019 0.1500 [0.0544; 0.3511]

Nakazaki_2013 0.0000 [0.0017; 0.3221]

Raldow_2013 0.2000 [0.0730; 0.4424]

Rava_2013 0.1500 [0.0767; 0.2728]

Number of studies combined: k = 12

proportion 95%-CI

Random effects model 0.1786 [0.1162; 0.2644]

Quantifying heterogeneity:

tau^2 = 0.3871; tau = 0.6222; I^2 = 79.3% [64.5%; 87.9%]; H = 2.20 [1.68; 2.88]

Test of heterogeneity:

Q d.f. p-value Test

53.13 11 < 0.0001 Wald-type

54.52 11 < 0.0001 Likelihood-Ratio

**Review: 24 Month Survival**

proportion 95%-CI

Ali_2017 0.0800 [0.0646; 0.0987]

Bowden_2019_Breast 0.4300 [0.2484; 0.6327]

Bowden_2019_Lung 0.0600 [0.0104; 0.2788]

Bowden_2019_Melanoma 0.1100 [0.0305; 0.3266]

Chang_2010_11-15_mets 0.2500 [0.1001; 0.4998]

Chang_2010_15_plus_mets 0.1000 [0.0344; 0.2573]

Ehrlich_2019 0.0750 [0.0289; 0.1811]

Izard_2019 0.2500 [0.1469; 0.3921]

Mizuno_2019 0.1500 [0.0544; 0.3511]

Nakazaki_2013 0.0000 [0.0017; 0.3221]

Raldow_2013 0.1500 [0.0462; 0.3915]

Rava_2013 0.1200 [0.0562; 0.2379]

Number of studies combined: k = 12

proportion 95%-CI

Random effects model 0.1283 [0.0804; 0.1984]

Quantifying heterogeneity:

tau^2 = 0.3757; tau = 0.6130; I^2 = 73.2% [52.3%; 84.9%]; H = 1.93 [1.45; 2.58]

Test of heterogeneity:

Q d.f. p-value Test

41.03 11 < 0.0001 Wald-type

40.37 11 < 0.0001 Likelihood-Ratio

- 1. **FUNNEL PLOT FOR RANDOM-EFFECTS META-ANALYSIS**


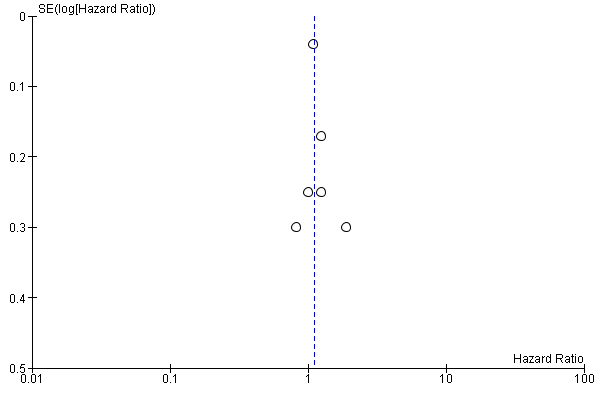

Supplement: Supplementary file 1 — Supplementary Material 1 [file 12885_2023_11452_MOESM1_ESM.docx]
